# Painful Abdominal Lump in the Pediatric Age Group: A Diagnostic Dilemma

**DOI:** 10.7759/cureus.13202

**Published:** 2021-02-07

**Authors:** Pranav Ajmera, Vikas Jadhav

**Affiliations:** 1 Radiology, Dr. D.Y. Patil Medical College, Hospital and Research Center, Pune, IND

**Keywords:** mesenteric cyst, imaging, cystic malformation, lymphangioma, child, ultrasound, computed tomography, gut signature, malignancy, neoplasia

## Abstract

Any cystic lesion occurring in the mesentery which may or may not extend into the retroperitoneum is referred to as a mesenteric cyst; they have an infrequent incidence rate in the pediatric age group. Definitive etiology of the cystic lymphatic malformations is still not known but there are multiple hypotheses. A young male child presented with acute onset abdominal pain and palpable intra-abdominal mass and ultrasonography revealed presence of two lesions, one of them as an encysted turbid fluid collection in the right lumbar region and the other as a dilated, tortuous, intercalated structure. On CT, the first one was identified definitively as a mesenteric cyst while the other as a possible neoplastic mass in close proximity to the first one. Histopathology confirmed the diagnosis as a cystic lymphatic malformation of the mesenteric cyst. The limited awareness of its existence along with its usually asymptomatic nature, are the likely reasons that it still remains an elusive diagnosis. Based on our case we discuss, the use of a multi-modality approach towards diagnosing cystic malformation disorders and how the use of MRI is under-utilised when it could prove decisive.

## Introduction

Any cystic lesion occurring in the mesentery which may or may not extend into the retoperitoneum is referred to as a mesenteric cyst. Reported cases in the literature of mesenteric cysts are far and few with a few reports stating their incidence between 1/10000 to 1/25000 in the pediatric age group and around 1/105000-250000 hospitalized adult surgical patients [1]. While these are found through all age groups, they are particularly common before 15 years of age [2,3]. Definitive etiology of the cystic lymphatic malformations is still not known, but the inability of the lymph nodes to communicate with the lymphatic system or venous system or occlusion of the lymphatic channels is a likely reason [2].

We report such a case where the likelihood of mesenteric cyst was kept foremost but the appearance was mimicking in places that of a malignancy. The limited awareness of its existence is one of the reasons that it still remains an elusive diagnosis with most cases being diagnosed incidentally. Herein, we have utilised this case to describe how the usage of a multi-modality approach involving ultrasound (US), computed tomography (CT) and magnetic resonance imaging (MRI) is highly effective in suspecting and confirming the diagnosis, and why MRI, if possible, must be carried out in all such cases [4].

## Case presentation

An eight-year-old male child presented to the paediatric department complaining of diffuse abdominal pain and abdominal distension since 15 days. On examination, there was a soft, palpable lump of size 5 x 4cm in the right lumbar and right iliac fossa region. He did not have any complaints associated with the passage of stools or episodes of vomiting. The child had an uncomplicated birth history and was developing normally for age; there was no family history of any such previous presentations. The referring doctor advised a US of abdomen and pelvis to establish the aetiology of the mass.

**Figure 1:**
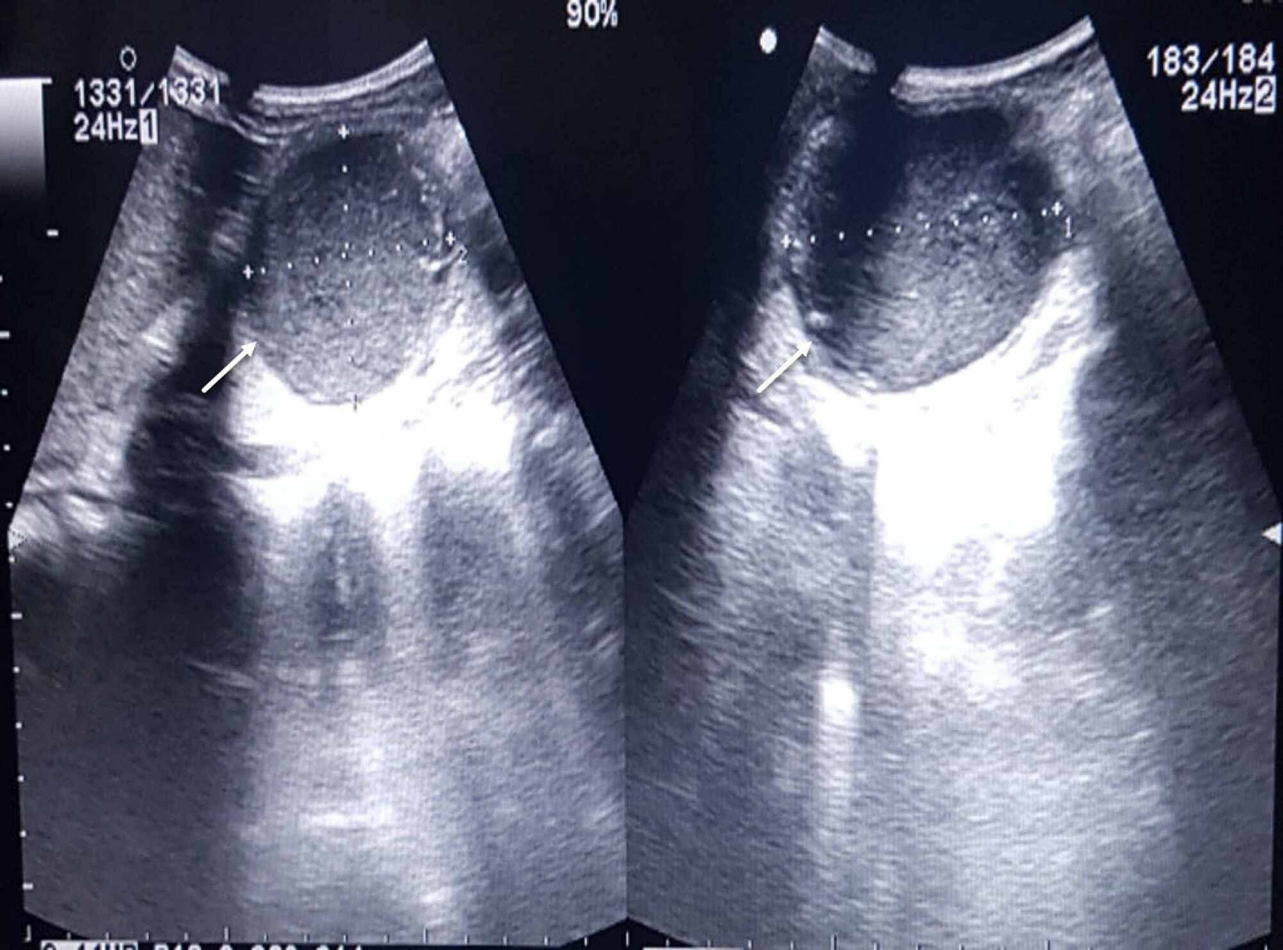
Ultrasonography by the convex probe, demonstrates the cystic lesion (White arrow), inferior to the liver.

An ultrasonographic evaluation revealed the presence of an encysted turbid fluid collection of size 71x81x56mm (161 cubic centimetres in volume) (Figure 1). The fluid was thick with a few foci of calcification and the walls had a thickness of 3mm and were not showing the gut signature. Immediately medial to this large collection were multiple tortuous, dilated, intercalated structures reaching up to the umbilicus. Likelihood of mesenteric cyst was raised for the turbid collection with inconclusive opinions about the tortuous, dilated structures (possibility of an extra-intestinal gastrointestinal stromal tumor (GIST) was considered).

**Figure 2:**
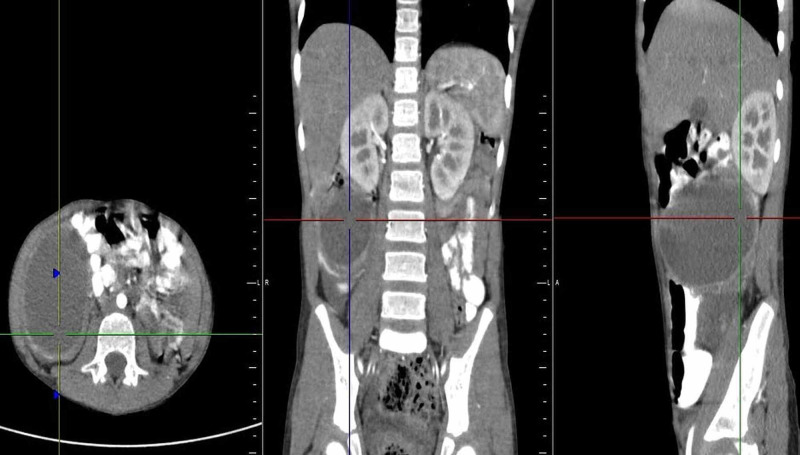
Contrast-enhanced CT image showing the size of the cyst and enhancement of the walls ( Center of the triangulation tool).

**Figure 3:**
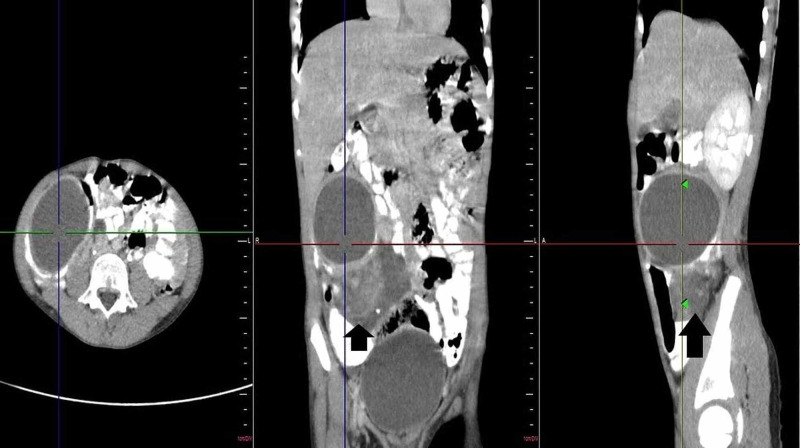
Contrast-enhanced CT image with the arrowhead directed towards the heterogeneously enhancing solid lesion.

For further assessment, a CT scan was advised, which revealed a well-defined, hypodense fluid density with a wall thickness of 2mm and size approximately 55mm (craniocaudal) x 80mm (anteroposterior) x 53mm (transverse) in the right lumbar and iliac region, between medial wall of the caecum and ascending colon laterally, and terminal ileum medially just beneath the anterior abdominal wall (Figure 2). This lesion was diagnosed as a mesenteric/duplication cyst with high protein content/haemorrhage/superimposed infection (multiple thick internal echoes with entrapped small echo-reflective calcific foci within cyst seen on US were not appreciated on CT study). The dilated, tortuous, structure reported on the US was visualised on CT as a solid-cystic lesion with irregular margins, located in the mesentery, in the right iliac fossa extending to umbilical region, measuring approximately 50mm (craniocaudal) x 36mm (anteroposterior) x 80mm (transverse) and showing heterogeneous post-contrast enhancement (Figure 3); enhancing mesenteric vessels were noted traversing through this lesion which also had an eccentrically placed appendix. This lesion was suspicious for a neoplastic mass like GIST or matted lymph nodal mass.

All lab parameters were within normal limits revealing haemoglobin as 12.7g/dl, total leucocyte count as 8300/µL, platelet count as 4,75,000/µL and an international normalized ratio (INR) of 1.01.

**Figure 4:**
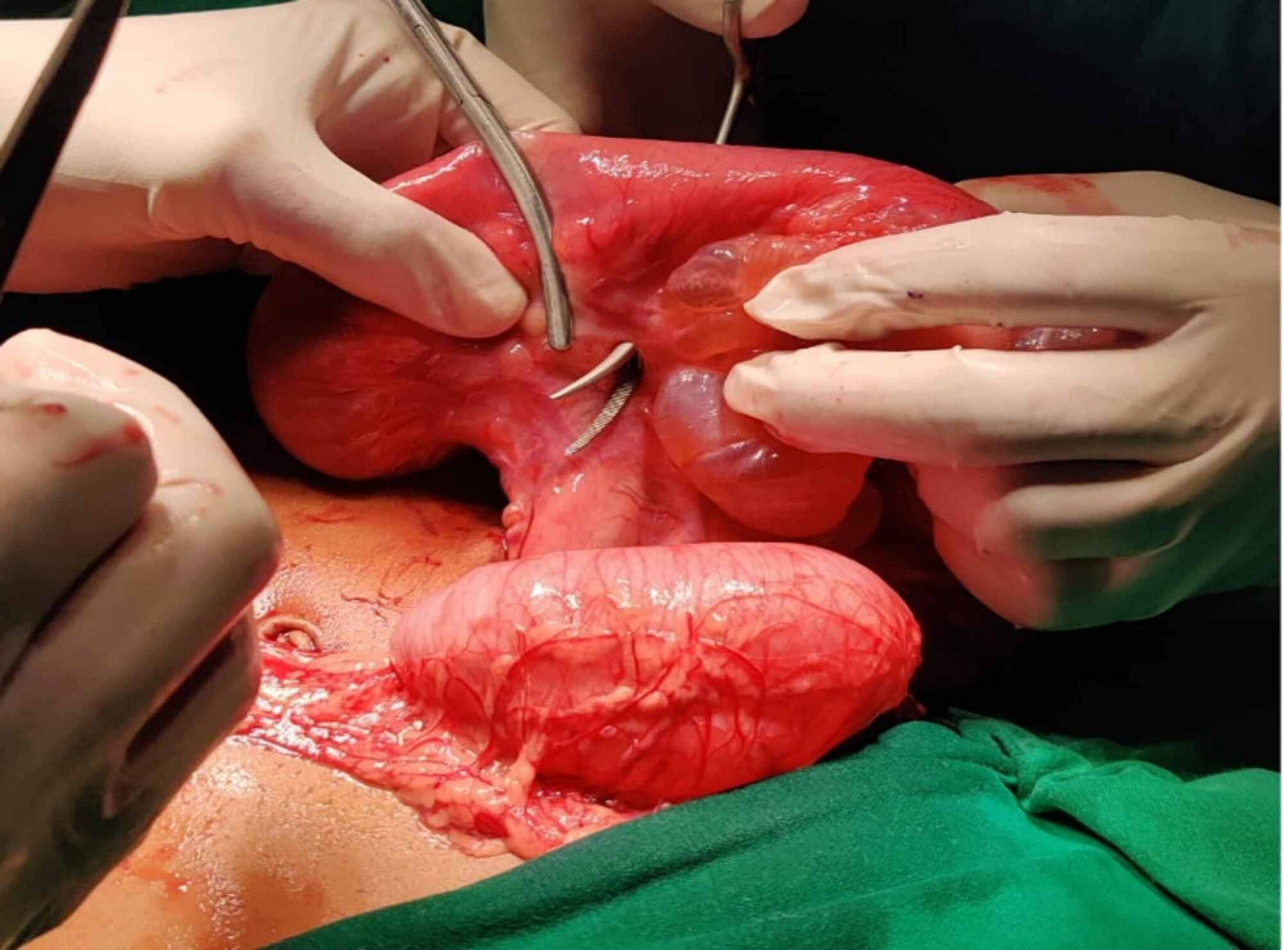
Intra-operative image of the cystic lesion located in the vicinity of the bowel.

**Figure 5:**
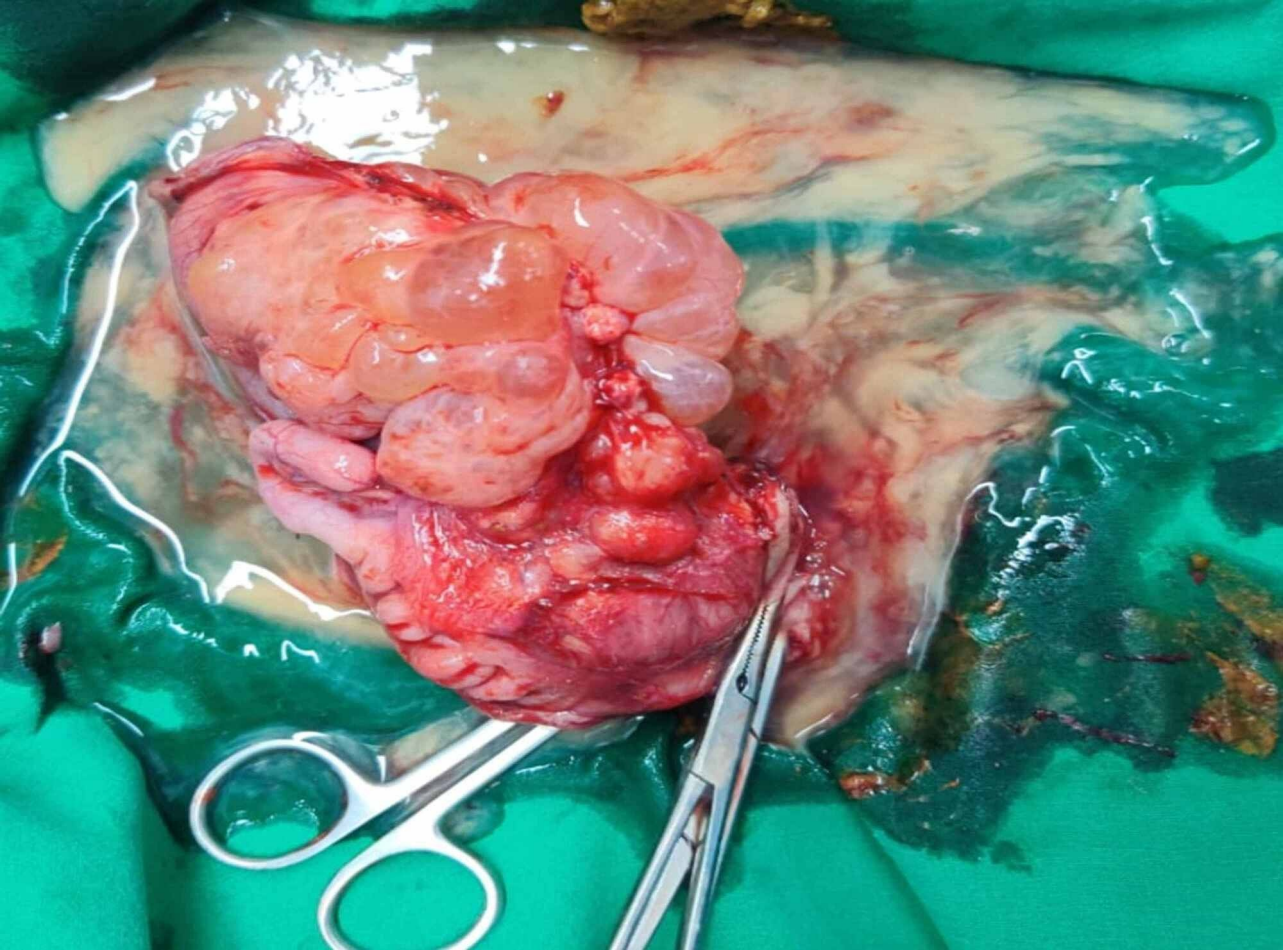
Surgically resected specimen of the bowel with the lesions, showing the thick turbid content of the mesenteric cyst.

**Figure 6:**
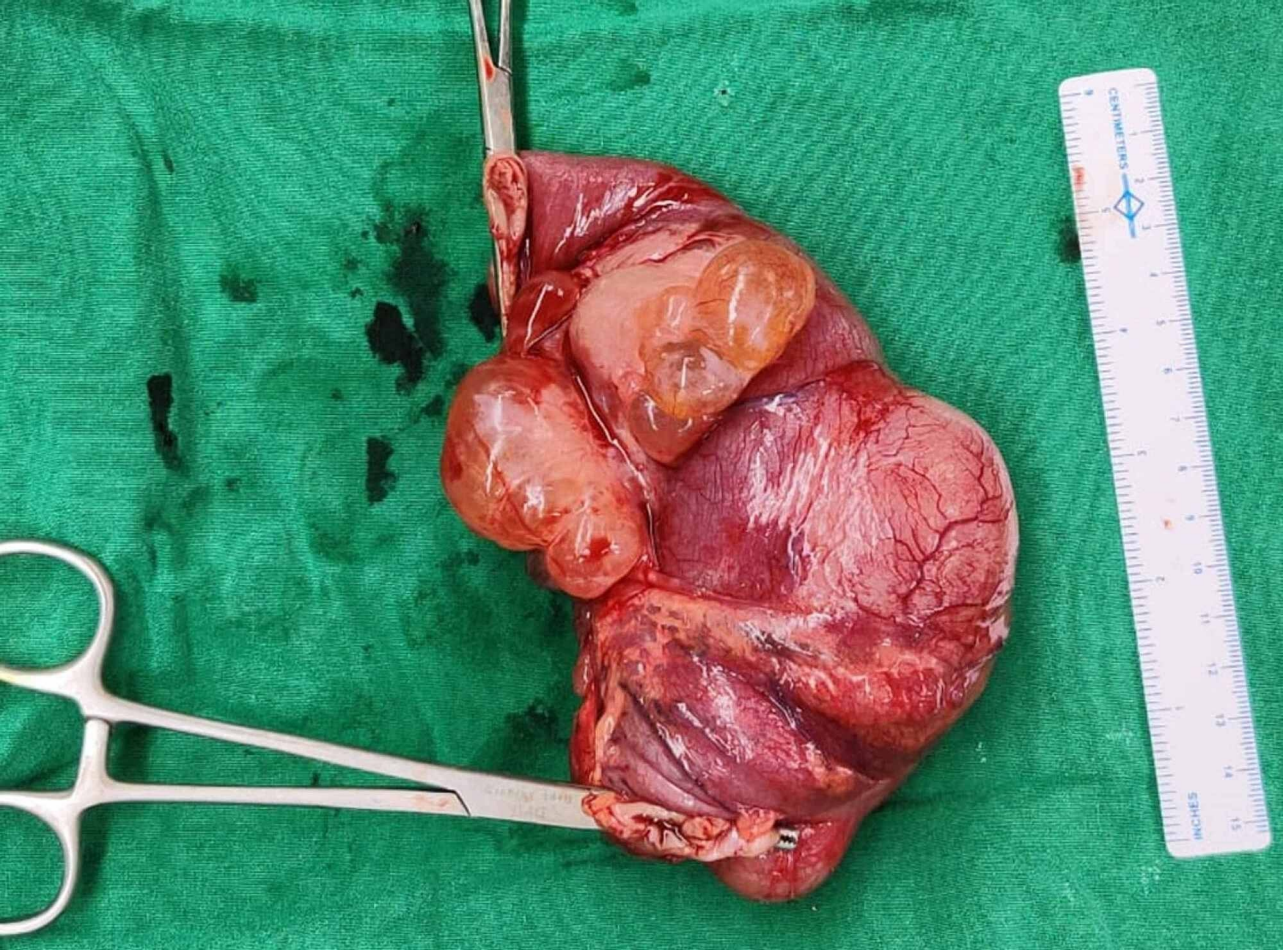
Surgically resected specimen of the bowel and lesions.

**Figure 7:**
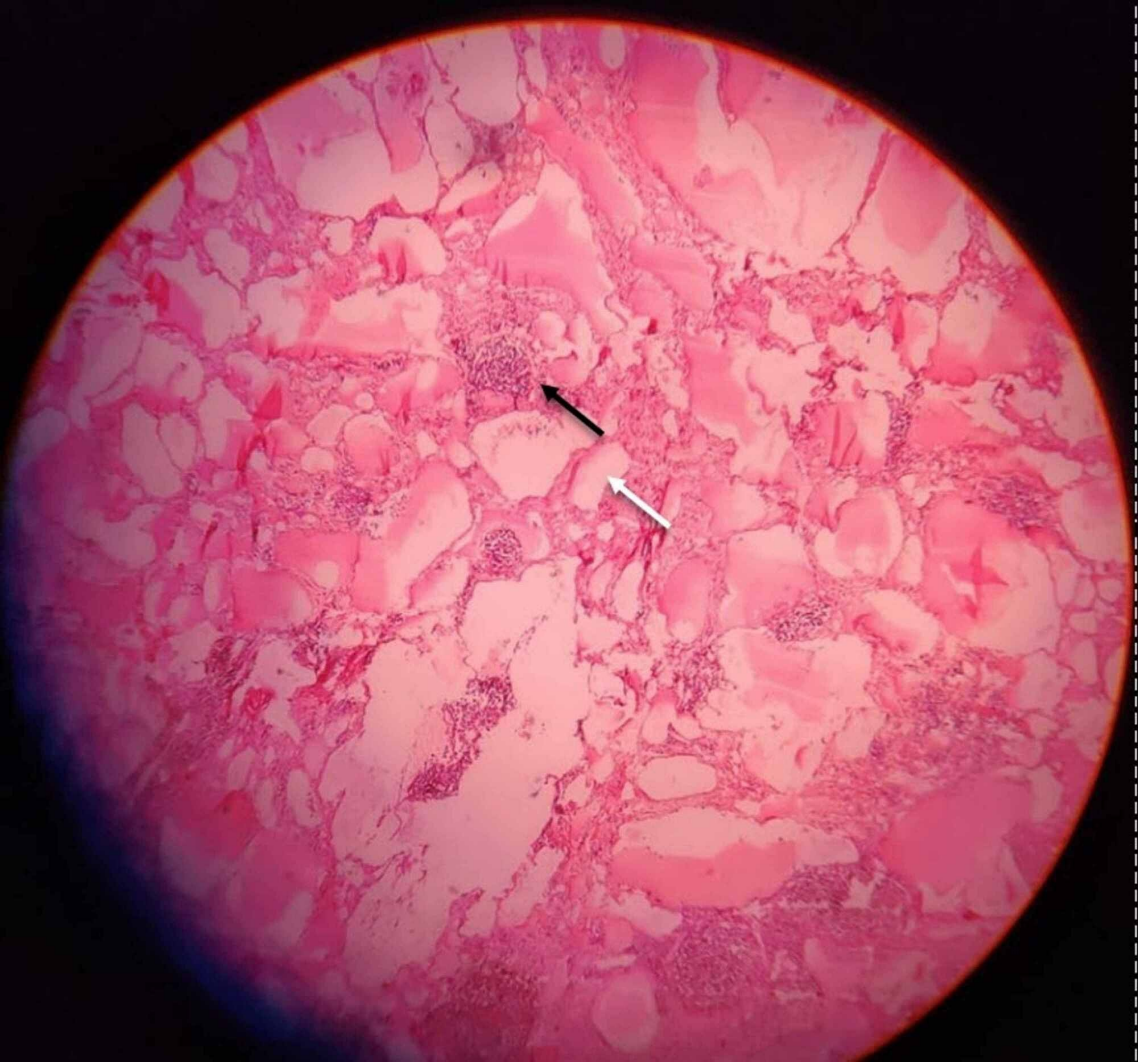
Photomicrograph of the lesion in scanner view, shows multiple dilated lymphatic channels (white arrow) with numerous lymphoid follicles (black arrow).

**Figure 8:**
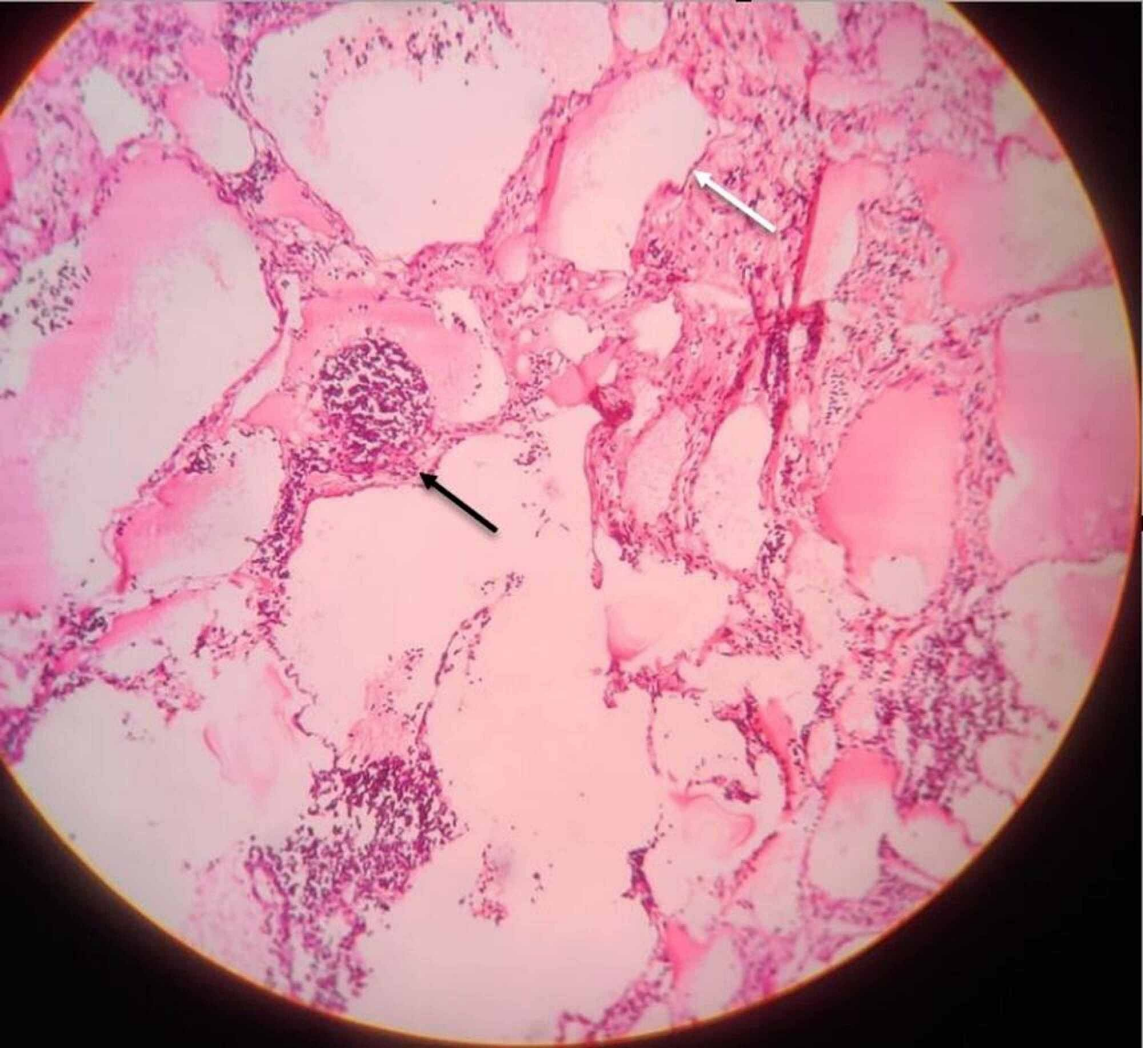
Photomicrograph of the lesion in low power view, shows dilated lymphatic channel (white arrow) and a few lymphoid follicles (black arrow).

**Figure 9:**
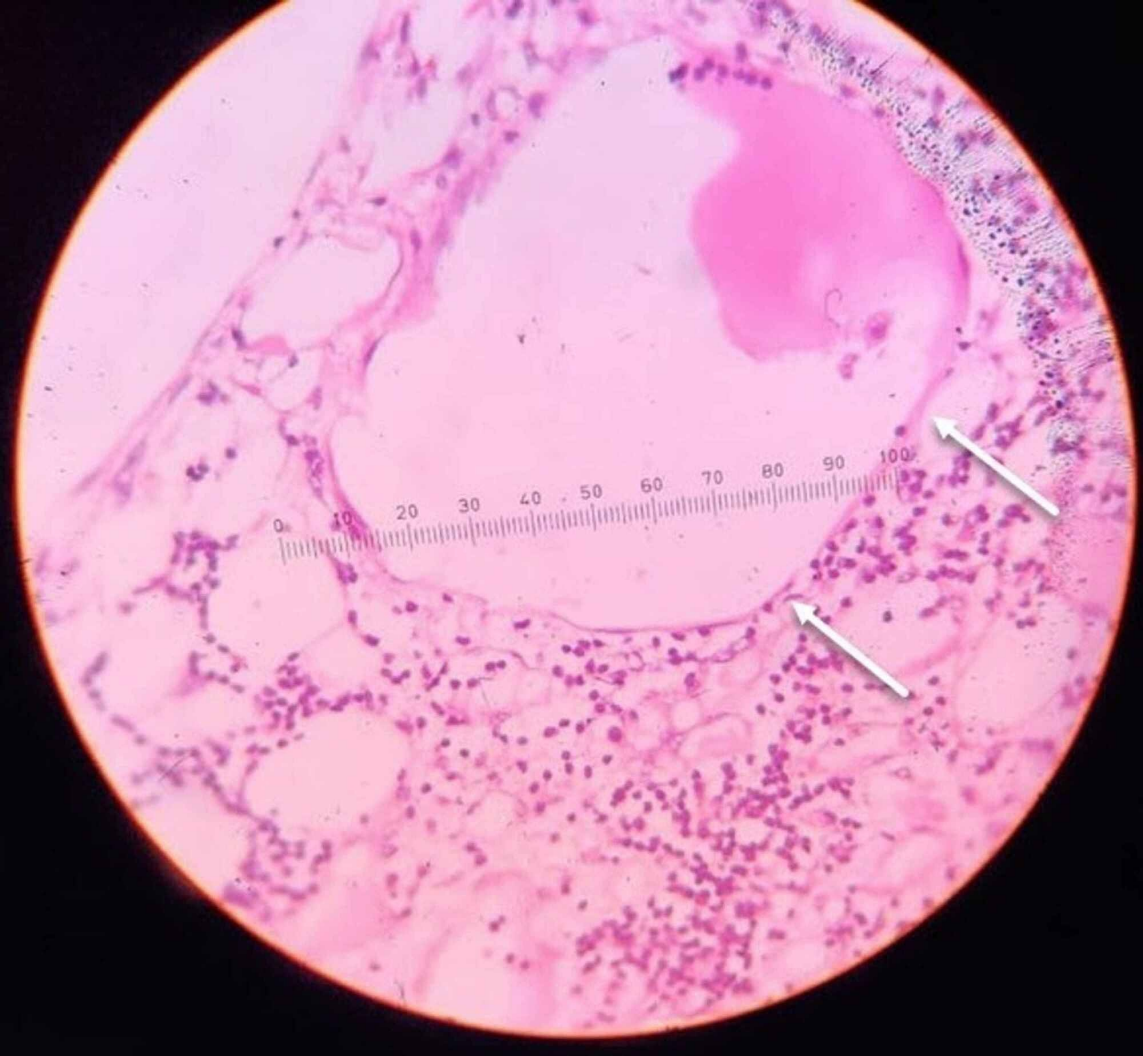
Photomicrograph of the lesion in high power view, shows dilated lymphatic channel with endothelial lining (white arrow).

The patient was planned for an exploratory laparotomy with the option for resection and anastomosis if needed. During laparotomy, the patient was found to have multiple, thin-walled, yellow coloured, translucent, encysted collections, adjacent to each other. Few enlarged paracolic lymph nodes were seen. Since these could not be removed, resection and anastomosis of the adjacent intestine were carried out (Figures 4-6) Appropriate samples were sent for histopathology (HPE), which revealed the larger cyst to have a cyst wall-like structure enclosing lymphatic spaces and small foci of lymphoid tissue. Examination of both the larger lesion and the smaller suspected neoplastic mass revealed, abundant dilated lymphatics with abundant scattered lymphoid tissue (Figures 7-9). Altogether the findings were suggestive of a mixed variety of common cystic malformation, as all of them were lymphatic cysts of varying sizes. Hence, the HPE examination excluded the possibility of any neoplastic mass which was considered as a second differential of the solid-cystic lesion on CT.

The patient became asymptomatic post the operation and had stable vitals. Both the one-month and six-month follow-up by clinical examination revealed a healthy, asymptomatic child.

## Discussion

First diagnosed by the anatomist Benevieni in 1507 during an autopsy, mesenteric cysts have been reported sporadically in literature. Since those times, a variety of mesenteric cysts have been documented, all of whom have similar pathogenesis but are varied based on their histo-pathological origin and structure. Frequently, they turn out to be an ectopic lymphatic tissue- lymphatic, chylous cysts.

One very commonly cited clinical classification groups mesenteric cysts under six broad categories, which include cysts of mesothelial origin, lymphatic origin, enteric origin, urogenital origin, mature cystic teratomas and non-pancreatic cysts [1]. The cysts of lymphatic origin are classified as per the ISSVA-2018 classification system into various types of lymphatic malformations, the most common of which is the common-cystic lymphatic malformation (CLM), which is further categorised based on size, those less than 1cm are referred to as microcystic and those more than 1cm are referred as macrocystic. The reported case was of the mixed cystic lymphatic malformation variety as on examination, there were multiple cystic lesions of sizes less than and more than 1cm. The exact incidence of cystic lymphatic malformations is difficult to determine due to the variation in terminology used for their reference in literature [5-8].

The cystic spaces have a lining of endothelium and the presence of lymphoid aggregated in the cyst wall. CLM usually contains serous, serosanguineous, or chylous fluid. The milky appearance of chylous fluid is due to abundant content of fat; variation in the appearance of the content of fluid is probably due to difference in degrees of lymph stasis and a variation in the number of communicating channels within the lymphatic system [6]. The size of the cyst and the patient's age can influence the symptoms of the patient [1,4]. The likely cause of acute presentation, in this case, was due to compression of the adjacent structures.

Together US and CT prove very effective in making the diagnosis [3]. In the reported case, the absence of a gut signature on US was useful in ruling out duplication cyst. This combined with the location of the lesion, narrowed down the differentials to a urachal cyst, GIST and mesenteric cyst. However, since typically urachal cysts are found between transverse fascia and the parietal peritoneum, the latter one was considered most likely; also, a tumor-like GIST is more common in middle-aged people. The CT appearance of a hypodense fluid collection, separate from the intestine, pancreas and ovaries, and the subsequent histopathological evaluation excluded the likelihood of malignant aetiology and reiterated the diagnosis as, cystic lymphatic malformation variety of mesenteric cyst. The lack of a gut signature is a critical finding and this can only be commented on US and is not appreciated on CT. 

While both US and CT were performed in the case, an MRI evaluation of abdomen could not be performed due to cost prohibitions; if indeed it had been, it would have been a very helpful adjunct. Gadolinium-enhanced MRI is useful in differentiating micro-cystic from macrocystic malformations prior to surgery, as lesions of small size and infiltrative nature are much better appreciated on contrast administration. MRI could have helped identify the contents as chylous or blood products. With chylous products, the MRI would have revealed the signal intensity drops out on fat-saturated images and chemical shift artefacts on opposed phase images [9-11].

Once diagnosis is certain then the management should preferably be surgical to avoid various future complications. Most commonly resected by surgical enucleation which also happens to be the preferred method of choice [1,9,10], but small-sized and uncomplicated cysts can be removed by laparoscopic approach [12]. In this case, due to the large size and close approximation to the adjacent structures, an open laparotomy followed by resection and anastomosis of adjacent bowel was carried out [2].

## Conclusions

To conclude, each modality adds on a layer of information towards our final diagnosis of mixed cystic lymphatic malformation; while the US allows for closer and dynamic visualisation of the cyst wall, CT allows us a better delineation of its extent and MRI as a final step allows us to comment upon the cyst contents, thus making ‘radiological biopsy’ possible that is the radiological description and diagnosis approaches such high levels of accuracy that it anticipates in advance the histopathological findings including a comment on the content of such lesions. It is thus advisable to systematically perform these modalities and give the treating surgeon an accurate diagnosis and description of what to expect and not leave them in the blind.
